# The effect of subjective social status on health-related quality of life decline in urban Chinese older adults: a four-year longitudinal study from Hong Kong

**DOI:** 10.1186/s12877-022-03314-x

**Published:** 2022-07-26

**Authors:** Timothy S. Sumerlin, Timothy C. Y. Kwok, William B. Goggins, Jinqiu Yuan, Elizabeth M. S. Kwong, Jason Leung, Jean H. Kim

**Affiliations:** 1 Jockey Club School of Public Health and Primary Care, Prince of Wales Hospital, The Chinese University of Hong Kong, Shatin, The New Territories Hong Kong, China; 2 Faculty of Medicine Department of Medicine and Therapeutics, Prince of Wales Hospital, The Chinese University of Hong Kong, Shatin, The New Territories Hong Kong, China; 3 Clinical Research Center, The Seventh Affiliated Hospital Sun Yat-sen University, Shenzhen, Guangdong China

**Keywords:** Subjective Social Status, Health-Related Quality of Life, Geriatric Social Medicine, Gerontology, China

## Abstract

**Background:**

Improving health-related quality of life (HRQOL) is becoming a major focus of old age care and social policy. Researchers have been increasingly examining subjective social status (SSS), one’s self-perceived social position, as a predictor of various health conditions. SSS encompasses not only concrete socio-economic (SES) factors but also intangible aspects of status. This study’s main objective was to examine the association between SSS and long-term change in HRQOL in older Chinese adults.

**Methods:**

A longitudinal Hong Kong study recruited 2934 community-dwelling adults (age > 65 years). Participants completed SF-12 physical health (PCS) and mental health (MCS) HRQOL scales. This study analyzed baseline SSS-Society (self-perceived social status within Hong Kong) and SSS-Community (self-perceived status within one’s own social network) as predictors of long-term HRQOL decline. After stratifying for sex, multiple-linear-regression was performed on 4-year follow-up SF-12 PCS and MCS scores after adjusting for baseline SF-12 scores, traditional SES indicators, demographic variables, clinical conditions, and lifestyle variables.

**Results:**

In the multivariable analyses, lower SSS-Society was associated with declines in MCS in males (β_standardized_ = 0.08, *p* = 0.001) and declines in PCS (β_standardized_ = 0.07, *p* = 0.006) and MCS (β_standardized_ = 0.12, *p* < 0.001) in females.

SSS-Community was associated with declines in PCS in males (β_standardized_ = 0.07, *p* = 0.005) and MCS in females (β_standardized_ = 0.14, *p* < 0.001).

**Conclusions:**

SSS may be a useful supplementary tool for predicting risk of long-term HRQOL decline in older Chinese adults. Strategies to reduce perceived social inequalities may improve HRQOL in older adults.

## Introduction

As a consequence of longer life spans and greater chronic disease burdens, there has been increasing emphasis on quality of life (QOL) as a health outcome and as a key consideration for healthcare decision-making for older adults [1–3]. Although the World Health Organization broadly defines QOL as “an individual’s perception of their position in life in the context of the culture and value systems in which they live and in relation to their goals, expectations, standards and concerns” [4], in health research, health-related quality of life (HRQOL) is commonly understood as the ability to partake in normal activities of daily living [5]. In recognition of the importance of HRQOL, policymakers in Eastern and Western countries and regions have been increasingly adopting healthy aging strategies to improve HRQOL of older adults. Policy action plans have included improved services for older adults, community-based interventions, policies to improve social participation, and health promotion for older adults [6, 7].

In older adults, the literature has shown a spectrum of factors that can affect HRQOL. In addition to a wide range of health conditions and impairments, psychosocial attributes such as social isolation as well as lifestyle habits were shown to have negative impacts on HRQOL [8–10]. A number of studies have also revealed that HRQOL is associated with socioeconomic status (SES) indictors, such as low income and low educational attainment in the general population and in different patient groups [8, 11–15]. It is theorized that those with higher SES have better health literacy, better access to healthcare, and greater social support, leading to improved health and HRQOL [16–18]. However, there are some limitations in using objective SES indicators. While SES factors like income and occupation are indicators of resources in a general working population, they are less meaningful in retired or disabled adults who may no longer have an income [19, 20]. Moreover, SES measures typically are measured at a single time point and do not capture the socioeconomic circumstances of one’s life course. Lastly, objective SES indicators may not fully capture the various aspects of higher social status such as community ties and esteem from peers that may influence health through various pathways. To overcome these limitations, researchers have increasingly been using other measures of social status [21].

Subjective social status (SSS), one’s self-perceived social status, is an alternative method of examining social status and contextual factors in relation to health. Respondents are asked to rank themselves on a visual analogue scale in comparison with others in their country or region (SSS-Society) and others in their self-designated community (SSS-Community). SSS-Society makes explicit reference to objective SES factors such as income, educational attainment, and occupation for the individual to assess how they compare with others in their country or region as a whole [22]. By contrast, SSS-Community does not make explicit reference to any SES factors. SSS-Community allows individuals to use their own subjective measures of social status and to define the community to which he/she belongs (e.g. church, neighborhood, workplace). SSS-Community has been previously noted to be conceptualized to include factors such as peer esteem and perceived social support [23]. Although SSS is asked at a single point in time, one purported advantage of SSS over traditional SES measures such as annual income, which can vary over time, is that SSS allows people to “cognitively average” various aspects of their social status over their life course [24]. In studies assessing both traditional SES indicators and SSS together, SSS was independently associated with health outcomes such as self-rated health and mortality, showing it to be independently a robust indicator of health [16, 21, 22]. Longitudinal studies have also noted robust associations between SSS with depression and cognitive decline, even after adjusting for SES indicators [25, 26].

Previous studies have established a statistically significant association between lower SSS and worse HRQOL and self-rated health, however, most of these studies utilized a cross-sectional study design [16, 18, 27–29], therefore, the direction of any significant associations could not be determined conclusively. Only two longitudinal studies assessing SSS and HRQOL have been conducted [30, 31]. A study conducted in the general adult population of Indonesia noted that baseline country-level SSS was a robust predictor of general self-rated health, physical functioning, and nurse-assessed general health after 7 years [31]. However, since the Indonesian study did not examine SSS-Community, many intangible aspects of social status may not have been captured in assessing effects of social status on long-term HRQOL. A study conducted in Germany assessed baseline SSS at the country and community level with physical and mental self-rated health at a year-2 follow-up [30]. The German study found that both SSS measures were associated with self-rated physical health at follow-up while only community-SSS was marginally associated with year-2 self-rated mental health after inclusion of objective SES measures. The authors of the German study called for further investigation in a non-European setting with greater economic inequality, as well as inclusion of health-related factors that may influence the relationship between SSS and HRQOL. Furthermore, since these prior studies were not focused on older adults, detailed information on chronic health conditions were not collected. Previous studies have found chronic health conditions and multi-morbidity to be associated with worse HRQOL in older adults [32, 33]. In order to examine the influence of SSS on long-term HRQOL declines in older adults, a longitudinal study of this population should ideally include not only socioeconomic indictors but also an array of common health conditions and relevant lifestyle factors.

The relationship between SSS and health have been previously noted to differ by sex [34–36]. A study of older adults in Japan, for instance, noted subjective status change had very different effects on mortality between males and females [34]. Another study exploring associations between SSS and various health outcomes between sexes found SSS to be associated with diabetes and HDL-cholesterol in females only [27]. Additionally, males and females may relate to and be affected by social status dimensions differently [36]. Therefore, previous studies have called for the need of future research to explore heterogeneity by sex when examining SSS and HRQOL [31].

Hong Kong, a special administrative region in southern China of 7.6 million people, possesses the highest income inequality among industrialized countries (GINI coefficient = 0.539) despite a very high per capita income [37]. Hong Kong also currently has the highest life expectancy in the world [38] and the population of those aged ≥65 are expected to nearly double by 2040 [39]. Similar to many other governments in the Asia region, the Hong Kong government formally made improving the QOL of older adults a strategic policy objective [7, 40]. In order to inform government aging-related social policies, this study aims to examine whether lower SSS is associated with greater long-term decline in physical and mental functioning HRQOL in older age Chinese adults, after adjusting for socioeconomic, clinical, and lifestyle factors.

## Methods

### Data collection

Beginning in 2001, 2000 male and 2000 female community-dwelling Chinese adults (age 65 years and above), enrolled in the Hong Kong Mr./Mrs. Os study, a longitudinal study examining osteoporosis and other non-communicable disease risk factors. The study data collection protocol is detailed previously and the baseline sample size was calculated based upon prevalence estimates of chronic health conditions [29]. At baseline, trained research staff used face-to-face interviews, collecting data about socio-demographic characteristics like sex and educational attainment. Additionally, history of starvation was used as a possible indicator of early life deprivation. At year-4 follow-up, 1559 males and 1519 females participated in the data collection (77% retention rate). Due to the questionable validity of self-recall among those with moderate and severe cognitive status, those with a Mini-Mental State Examination score < 20 were removed from our sample population, resulting in 1542 males and 1392 females remaining for analysis. In addition to the above variables, the baseline study also collected nutrition and self-reported health data, anthropometric information and clinical assessments on various health-related factors. For this analysis, we included data on physical health conditions (history of stroke, diabetes, chronic obstructive pulmonary disease, osteoporosis, cardiovascular conditions, thyroid conditions), smoking status, and alcohol consumption. Physical activity levels were assessed using Physical Activity Scale for Elderly (scored 0–793 points) [41]. Due to the lack of recommended cut-offs, respondents whose scores were less than the IQR for our sample were coded as having low Physical Activity Scale for Elderly scores (0 = Scale score within or higher than IQR, 1 = Score below the IQR).

### Subjective social status

This study’s main predictor of interest, SSS, was measured using the visual analogue MacArthur Scale, depicting a ladder with 10 rungs (scored 1–10). For SSS-Society, respondents were asked: “This ladder shows where people stand in Hong Kong. At the top are those who are best off – those who have the most money, best education, and most respected jobs while those at the bottom are those who are worst off. Please place an “X” where you would place yourself relative to others in Hong Kong”. The SSS-Community item asks respondents: “Think of this ladder as representing where people stand in their communities. Define community in whatever way is meaningful to you. At the top are those who have the highest standing in your community while those at the bottom have the lowest” [22, 42]. In past studies, both scales have shown moderate to good stability test-retest reliability and clear construct validity [43–45].

### Health-related quality of life

Health-related quality of life, the outcome variable, was measured at baseline and year-4 using a validated Chinese version of the SF-12 [46]. The SF-12 measure is a shortened version of the SF-36, that also measures the same eight domains: physical functioning, physical role limitations, bodily pain, general health perceptions, energy/vitality, social functioning, emotional role limitations, and mental health. The resulting two scores, the physical component score (PCS), Cronbach’s alpha = 0.71, and mental component score (MCS), Cronbach’s alpha = 0.74. The PCS and MCS scores each range from 0 to 100 (higher score indicating better health-related quality of life). In this study, the PCS and MCS were both assessed as a continuous variable. Ethics approval was obtained from the sponsoring university’s clinical ethics committee and all ethical safeguards in accordance with the Declaration of Helsinki were met.

### Statistical analysis

Due to previously noted differences in associations between SSS and health between sexes and to facilitate comparison in previous studies [24, 34–36], all analyses were stratified by sex. SSS-Society, SSS-Community, sociodemographic, clinical, and lifestyle variables were first individually tested for associations with year 4 follow-up PCS and MCS scores using linear regression models, only adjusting for baseline PCS and MCS scores (‘bivariable baseline-adjusted models’). Variables which produced a *p*-value ≤0.20 in the bi-variable model were retained for the multivariable model. For the final multi-variable models, backwards selection linear regression was performed and variables producing a p-value > 0.05 were dropped one-by-one, however, if they changed the main effect of interest by more than 10%, they were retained in the model. The two main predictor variables of interest, SSS-Society and SSS-Community, baseline PCS and baseline MCS, as well as one SES factor, educational attainment, were all forced into the final model.

We checked the assumptions of linear regression for normality and heteroscedacity of the residuals using residuals plots and checked for collinearity by looking at the variance inflation factor.

## Results

Table 1 describes the background characteristics of the study sample who returned for year-4 follow up (*n* = 2934). There were more males than females reporting for year-4 follow-up, with 73.6% of the sample between the ages of 65–74 at baseline. Approximately half of the respondents attended at least some primary school, but over one-third of females (34.0%) received no schooling compared to just 4.8% of males. Just 6.8% of males were widowed compared to 38.1% of females. Additionally, females were more likely to be living alone, less likely to have smoked or currently drink alcohol, and slightly less likely to have experienced a period of starvation in their life (*p* < 0.05). Males had higher physical activity levels and higher cognitive functioning scores, but lower scores than females in SSS-Community and slightly lower scores than females in SSS-Society (p < 0.05). Due to the marked differences between male and female respondents, all analyses were stratified by sex.

**Table 1:** Baseline background information of the study sample

Variables	Male (*n* = 1542) % (n)	Female (*n* = 1392) % (n)	*p*-value	All (*n* = 2934) % (n)
Demographic & background attributes				
Age			0.214	
65–74 years old	74.5% (1149)	72.5% (1009)		73.6% (2158)
75+ years old	25.5% (393)	27.5% (383)		26.4% (776)
Educational level			< 0.001	
No Schooling	4.7% (73)	30.0% (418)		16.7% (491)
At least some primary school	53.6% (827)	49.9% (694)		51.8% (1521)
At least some secondary school	26.9% (415)	13.1% (182)		20.3% (597)
At least some university/college	14.7% (227)	7.0% (98)		11.1% (325)
Marital Status			< 0.001	
Currently married	89.8% (1384)	55.4% (793)		74.2% (2177)
Never married	1.6% (24)	2.0% (28)		1.8% (52)
Divorced/Separated	1.8% (29)	2.9% (40)		2.3% (69)
Widowed	6.8% (105)	38.1% (531)		21.7% (636)
Currently living alone	6.2% (95)	19.0% (265)	< 0.001	12.3% (360)
Lifetime ever smoker	62.1% (958)	8.7% (121)	< 0.001	36.8% (1079)
Currently consumes alcohol	24.6% (379)	2.8% (39)	< 0.001	14.3% (418)
History of starvation in lifetime	61.4% (908)	55.9% (711)	0.004	58.9% (1619)
Body mass index Mean (SD)	23.5 (3.0)	23.9 (3.3)	0.003	23.7 (3.2)
Health Conditions				
Diabetes	13.9% (215)	13.1% (183)	0.529	13.6% (398)
Hyperthyroidism	1.5% (23)	4.9% (68)	< 0.001	3.1% (91)
Hypothyroidism	0.8% (12)	2.4% (46)	< 0.001	1.6% (46)
Osteoporosis	2.9% (44)	8.5% (118)	< 0.001	5.5% (162)
History of stroke	4.5% (69)	3.2% (45)	0.082	3.9% (114)
Hypertension (Systolic BP > 140, diastolic BP > 90)	41.4% (638)	43.9% (611)	0.168	42.6% (1249)
CVD Conditions ^a^	17.6% (272)	16.6% (231)	0.453	17.1% (503)
Chronic obstructive pulmonary disease	9.9% (153)	4.7% (66)	< 0.001	7.5% (219)
Baseline instruments scale scores Mean (SD)				
Physical Activity Scale for Elderly (PASE) ^b^	100.0 (50.8)	87.6 (33.8)	0.023	94.6 (44.1)
SF-12 Physical Functioning Score ^c^	51.1 (7.1)	47.2 (8.6)	< 0.001	49.2 (8.1)
SF-12 Mental Functioning Score ^c^	56.1 (6.5)	55.3 (7.5)	0.003	55.7 (7.0)
SSS-Society ^d^	4.5 (1.8)	4.7 (1.8)	0.014	4.6 (1.8)
SSS-Community ^d^	6.4 (2.2)	7.3 (2.0)	< 0.001	6.8 (2.2)
Year 4 instruments scale scores Mean(SD)				
SF-12 Physical Functioning Score ^c^	50.0 (7.7)	46.2 (9.5)	< 0.001	48.2 (8.8)
SF-12 Mental Functioning Score ^c^	57.3 (6.4)	56.5 (8.4)	0.003	56.9 (7.4)

x^2^ *p* values are shown for categorical variables while t-test *p* values are shown for continuous variables. ^a^ CVD (cardiovascular diseases) conditions (congestive heart failure, angina, myocardial infarction). ^b^ PASE Score (scores range from 0 to 793). ^c^ SF-12 Physical Functioning Score and SF-12 Mental Functioning Score (scores range from 0 to 100 with higher score reflecting better functioning). ^d^ SSS, subjective social status; SSS-Society & SSS-Community (scores range from 1 to 10)

The linear regression analyses of year-4 PCS are shown in Table 2 (male) and Table 3 (female). In the baseline PCS-adjusted bivariable models, apart from a lower baseline PCS score, hypertension and slower walking speed were associated with a lower year-4 PCS score (*p* < 0.05) in both males and females. Lower year-4 PCS score was also associated with lower SSS-Community score, older age, and a lower Physical Activity Scale for Elderly score in males and associated with lower SSS-Society, lower education, living alone status, diabetes, CVD conditions, smoking history and obesity in females.

**Table 2:** Linear regression model of year-4 physical component score among males (n = 1542)

	Bivariable model (PCS baseline)		SSS-Society model		SSS-Community model			
Factors	B Coeff (95% CI)	*p*-value	B Coeff (95% CI)	Std. Coeff	*p*-value	B Coeff (95% CI)	Std. Coeff	*p*-value
SSS-Society	0.11 (− 0.08, 0.31)	0.260	**0.09 (− 0.11, 0.29)**	**0.02**	**0.389**	–	–	–
SSS-Community	0.23 (0.07, 0.40)	0.006	–	–	–	**0.24 (0.07, 0.40)**	**0.07**	**0.005**
Baseline PCS	0.42 (0.37, 0.47)	< 0.001	**0.38 (0.33, 0.43)**	**0.35**	**< 0.001**	**0.37 (0.32, 0.42)**	**0.35**	**< 0.001**
Demographics								
Age	−0.12 (− 0.19, − 0.04)	0.002	− 0.03 (− 0.11, 0.05)	−0.02	0.442	−0.04 (− 0.12, 0.04)	−0.02	0.326
Education		0.098			**0.508**			**0.523**
No schooling	1.00		**1.00**			**1.00**		
At least some primary school	0.53 (− 1.16, 2.22)		**0.22 (− 1.45, 1.90)**	**0.02**		**0.08 (− 1.60, 1.76)**	**0.01**	
At least some secondary school	1.42 (−0.33, 3.18)		**0.84 (− 0.91, 2.58)**	**0.05**		**0.69 (− 1.06, 2.44)**	**0.04**	
At least some university/ college	1.28 (−0.58, 3.14)		**0.44 (− 1.44, 2.31)**	**0.02**		**0.21 (−1.66, 2.08)**	**0.01**	
Currently married	−0.73 (− 1.89, 0.44)	0.220	–	–	–	–	–	–
Well-being factors								
PASE Score (<IQR)	− 1.49 (− 2.30, − 0.69)	< 0.001	**− 1.17 (− 1.98, − 0.36)**	**−0.07**	**0.005**	**− 1.23 (− 2.04, − 0.43)**	**−0.07**	**0.003**
Currently living alone	−0.49 (− 1.95, 0.98)	0.514	–	–	–	–	–	–
Medical history								
Diabetes	−0.51 (− 1.53, 0.51)	0.325	–	–	–	–	–	–
Hyperthyroidism	−2.35 (− 5.25, 0.56)	0.114	− 2.15 (− 5.03, 0.72)	−0.03	0.142	− 2.12 (− 4.99, 0.75)	− 0.03	0.147
Hypothyroidism	1.07 (− 2.94, 5.08)	0.601	–	–	–	–	–	–
Osteoporosis	0.70 (− 1.42, 2.83)	0.516	–	–	–	–	–	–
History of stroke	− 1.58 (− 3.29, 0.13)	0.070	−0.48 (− 2.19, 1.23)	−0.01	0.584	−0.60 (− 2.31, 1.10)	−0.02	0.488
Hypertension	− 1.37 (− 2.08, − 0.66)	< 0.001	**−1.15 (− 1.86, − 0.44)**	**−0.07**	**0.002**	**−1.14 (− 1.85, − 0.43)**	**−0.07**	**0.002**
CVD conditions	−0.66 (− 1.59, 0.27)	0.161	− 0.19 (− 1.12, 0.75)	−0.01	0.697	−0.18 (− 1.11, 0.75)	−0.01	0.701
COPD	−0.97 -2.16, 0.21)	0.108	−0.75 (− 1.92, 0.42)	− 0.03	0.211	− 0.78 (− 1.95, 0.39)	−0.03	0.190
Current alcohol use	0.57 (−0.26, 1.38)	0.177	0.14 (− 0.68, 0.96)	0.01	0.734	0.17 (−0.65, 0.99)	0.01	0.688
Ever smoked	−0.00 (− 0.73, 0.73)	0.996	–	–	–	–	–	–
History of starvation	−0.15 (− 0.89, 0.59)	0.692	–	–	–	–	–	–
BMI > = 30	−0.01 (− 2.48, 2.47)	0.995	–	–	–	–	–	–
Walking speed (m/sec)	5.55 (3.94, 7.17)	< 0.001	**4.86 (3.18, 6.55)**	**0.14**	**< 0.001**	**4.72 (3.06, 6.39)**	**0.14**	**< 0.001**

*SSS* Subjective social status, *MCS* Mental Component Score, *PASE* Physical Activity Scale for Elderly, *IQR* Interquartile range, *CVD* Cardiovascular diseases, *COPD* Chronic obstructive pulmonary disease, *BMI* Body mass index. Values in bold represent those variables retained in the final model. Values not bolded but shown in the final model column represent their value before being removed from the model

SSS-Society: VIF range 1.015 to 1.116, R square 0.187

SSS-Community: VIF range 1.016 to 1.115, R square 0.190

**Table 3:** Linear regression model of year-4 physical component score among females (n = 1392)

	Bivariable model (PCS baseline)		SSS-Society model		SSS-Community model			
Factors	B Coeff (95% CI)	*p*-value	B Coeff (95% CI)	Std. Coeff	*p*-value	B Coeff (95% CI)	Std. Coeff	*p*-value
SSS-Society	0.45 (0.19, 0.71)	0.001	**0.36 (0.11, 0.62)**	**0.07**	**0.006**	–	–	–
SSS-Community	0.20 (− 0.03, 0.43)	0.081	–	–	–	**0.21 (− 0.02, 0.43)**	**0.04**	**0.072**
Baseline PCS	0.42 (0.37, 0.47)	< 0.001	**0.35 (0.30, 0.41)**	**0.32**	**< 0.001**	**0.35 (0.30, 0.41)**	**0.32**	**< 0.001**
Demographics								
Age	− 0.03 (− 0.12, 0.07)	0.554	**–**	**–**	**–**			
Education		0.012			**0.053**			**0.027**
No schooling	1.00		**1.00**			**1.00**		
At least some primary school	1.06 (0.00, 2.13)		**0.90 (−0.16, 1.96)**	**0.05**		**1.00 (− 0.04, 2.05)**	**0.05**	
At least some secondary school	2.50 (0.97, 4.02)		**1.48 (−0.08, 3.03)**	**0.05**		**1.85 (0.35, 3.36)**	**0.07**	
At least some university/ college	0.40 (−1.53, 2.34)		**−0.94 (−2.90, 1.03)**	**− 0.03**		**− 0.61 (− 2.53, 1.31)**	**−0.02**	
Currently married	−0.84 (− 1.77, 0.10)	0.078	0.01 (− 1.11, 1.13)	0.00	0.983	0.13 (−0.97, 1.22)	0.01	0.821
Well-being factors								
PASE Score (IQR)	−0.11 (−1.20, 0.97)	0.836	–	–	–	–	–	–
Currently living alone	−1.40 (−2.57, − 0.22)	0.020	−1.17 (− 2.34, 0.01)	−0.05	0.052	−1.09 (− 2.25, 0.07)	− 0.05	0.065
Medical history								
Diabetes	−2.74 (−4.10, − 1.38)	< 0.001	**−2.09 (−3.45, − 0.73)**	**− 0.08**	**0.003**	**−2.42 (− 3.77, − 1.08)**	**− 0.09**	**< 0.001**
Hyperthyroidism	−0.12 (− 2.26, 2.02)	0.913	–	–	–	–	–	–
Hypothyroidism	−1.00 (− 4.00, 1.99)	0.512	–	–	–	–	–	–
Osteoporosis	−0.67 (− 2.33, 1.00)	0.431	–	–	–	–	–	–
History of stroke	−1.56 (− 4.18, 1.06)	0.242	–	–	–	–	–	–
Hypertension	−0.94 (− 1.88, − 0.01)	0.047	−0.28 (− 1.23, 0.67)	−0.02	0.566	−0.32 (− 1.26, 0.63)	0.02	0.512
CVD conditions	−2.79 (− 4.04, − 1.55)	< 0.001	**−2.30 (− 3.54, − 1.06)**	**−0.09**	**< 0.001**	**−2.26 (− 3.49, − 1.03)**	**−0.09**	**< 0.001**
COPD	0.07 (− 2.11, 2.26)	0.948	–	–	–	–	–	–
Current alcohol use	−0.05 (− 2.85, 2.76)	0.973	–	–	–	–	–	–
Ever smoked	− 1.76 (− 3.39, − 0.12)	0.036	−0.71 (− 2.36, 0.95)	− 0.02	0.401	− 0.04 (− 1.02, 0.95)	−0.00	0.943
History of starvation	−0.55 (− 1.53, 0.43)	0.273	–	–	–	–	–	–
BMI > = 30	−5.05 (− 7.54, − 2.55)	< 0.001	**−3.98 (− 6.45, − 1.51)**	**− 0.08**	**0.002**	**−4.24(− 6.72, − 1.76)**	**− 0.08**	**0.001**
Walking speed (m/sec)	7.35 (5.02, 9.68)	< 0.001	**6.52 (4.12, 8.92)**	**0.14**	**< 0.001**	**6.64 (4.28, 9.00)**	**0.14**	**< 0.001**

*SSS* Subjective social status, *MCS* Mental Component Score, *PASE* Physical Activity Scale for Elderly, *IQR* Interquartile range, *CVD* Cardiovascular diseases, *COPD* Chronic obstructive pulmonary disease, *BMI* Body mass index. Values in bold represent those variables retained in the final model. Values not bolded but shown in the final model column represent their value before being removed from the model

SSS-Society: VIF range 1.010 to 1.116, R square 0.192

SSS-Community: VIF range 1.010 to 1.119, R square 0.189

The multivariable models revealed that for males lower SSS-Community was associated with decline in year-4 PCS (β_unstandardized_ = 0.24, 95% CI: 0.07, 0.40; β_standardized_ = 0.07) while SSS-Society did not show a statistically significant association. By contrast, in older females lower SSS-Society was a statistically significant predictor of decline in year-4 PCS (β_unstandardized_ = 0.36, 95% CI: 0.11, 0.62; β_standardized_ = 0.07) while SSS-Community was not. In females, SSS-Community model, those with no education had significantly higher year-4 PCS compared those with least some secondary school education. Across all PCS models, lower baseline PCS score, and slower walking speed were associated with decline in year-4 PCS.

The linear regression analyses of year-4 MCS are shown in Table 4 (male) and Table 5 (female). In the baseline MCS-adjusted bivariable models, apart from a lower baseline MCS, lower SSS-Society, hypothyroidism, and history of stroke were associated with a lower year-4 MCS (*p* < 0.05) in males and females. Osteoporosis was associated with lower year-4 MCS, in males only. Among females, lower SSS-Community, being currently not married, currently living alone, hyperthyroidism, CVD conditions, and having a history of starvation were associated with lower year-4 MCS.

**Table 4:** Linear regression model of year-4 mental component score among males (*n* = 1542)

	Bivariable model (MCS baseline)		SSS-Society model		SSS-Community model			
Factors	B Coeff (95% CI)	*p*-value	B Coeff (95% CI)	Std. Coeff	*p*-value	B Coeff (95% CI)	Std. Coeff	*p*-value
SSS-Society	0.27 (0.10, 0.43)	0.002	**0.29 (0.12, 0.47)**	**0.08**	**0.001**	–	–	–
SSS-Community	0.13 (− 0.01, 0.27)	0.068	–	–	–	**0.12 (− 0.02, 0.26)**	**0.04**	**0.092**
Baseline MCS	0.32 (0.27, 0.36)	< 0.001	**0.31 (0.27, 0.36)**	**0.32**	**< 0.001**	**0.29 (0.24, 0.33)**	**0.30**	**< 0.001**
Demographics								
Age	0.04 (− 0.03, 0.10)	0.297	–	–	–	–	–	–
Education		0.418			**0.310**			**0.414**
No schooling	1.00		**1.00**			**1.00**		
At least some primary school	0.86 (− 0.59, 2.31)		**0.84 (− 0.60, 2.29)**	**0.07**		**0.51 (− 0.92, 1.95)**	**0.04**	
At least some secondary school	0.46 (− 1.05, 1.97)		**0.24 (− 1.27, 1.75)**	**0.02**		**−0.06 (− 1.56, 1.44)**	**− 0.00**	
At least some university/ college	1.04 (− 0.57, 2.64)		**0.69 (−0.92, 2.30)**	**0.04**		**0.49 (− 1.11, 2.10)**	**0.03**	
Currently married	−0.94 (− 1.94, 0.05)	0.064	− 0.76 (− 1.75, 0.24)	−0.04	0.136	−0.88 (− 1.86, 0.14)	−0.04	0.091
Well-being factors								
PASE Score (IQR)	0.21 (−0.48, 0.91)	0.550	–	–	–	–	–	–
Currently living alone	−1.16 (− 2.42, 0.10)	0.071	−0.32 (− 1.85, 1.21)	−0.01	0.681	−0.18 (− 1.70, 1.35)	−0.01	0.821
Medical history								
Diabetes	−0.18 (− 1.05, 0.70)	0.695	–	–	–	–	–	–
Hyperthyroidism	− 1.99 (−4.49, 0.51)	0.118	− 1.06 (−3.76, 1.63)	−0.02	0.438	−1.14 (− 3.84,1.56)	− 0.02	0.406
Hypothyroidism	−4.75 (−8.19, − 1.31)	0.007	**− 4.85 (− 8.27, − 1.43)**	**−0.07**	**0.005**	**−4.41 (−7.90, − 0.92)**	**−0.06**	**0.013**
Osteoporosis	−1.92 (− 3.74, − 0.11)	0.038	−1.30 (− 3.13, 0.53)	− 0.03	0.162	−1.49 (− 3.31, 0.34)	−0.04	0.110
History of stroke	− 2.22 (− 3.68, − 0.75)	0.003	**− 2.23 (− 3.68, − 0.78)**	**−0.07**	**0.003**	**−1.84 (−3.28, − 0.40)**	**−0.06**	**0.012**
Hypertension	0.00 (−0.61, 0.62)	0.996	–	–	–	–	–	–
CVD conditions	−0.18 (− 0.98, 0.62)	0.659	–	–	–	–	–	–
COPD	−0.51 (− 1.52, 0.51)	0.328	–	–	–	–	–	–
Current alcohol use	0.36 (− 0.35, 1.06)	0.317	–	–	–	–	–	–
Ever smoked	0.09 (−0.54, 0.71)	0.784	–	–	–	–	–	–
History of starvation	−0.56 (− 1.18, 0.06)	0.076	− 0.55 (− 1.17, 0.08)	−0.04	0.087	**−0.69 (−1.31, − 0.06)**	**−0.05**	**0.031**
BMI > = 30	0.67 (− 1.46, 2.80)	0.536	–	–	–	–	–	–
Walking speed (m/sec)	0.24 (− 1.14, 1.62)	0.736	–	–	–	–	–	–

*SSS* Subjective social status, *MCS* Mental Component Score, *PASE* Physical Activity Scale for Elderly, *IQR* Interquartile range, *CVD* Cardiovascular diseases, *COPD* Chronic obstructive pulmonary disease, *BMI* Body mass index. Values in bold represent those variables retained in the final model. Values not bolded but shown in the final model column represent their value before being removed from the model

SSS-Society: VIF range 1.001 to 1.074, R square 0.121

SSS-Community: VIF range 1.004 to 1.049, R square 0.105

**Table 5:** Linear regression model of year-4 mental component score among females (*n* = 1392)

	Bivariable model (MCS baseline)		SSS-Society model			SSS-Community model		
Factors	B Coeff (95% CI)	*p*-value	B Coeff (95% CI)	Std. Coeff	*p*-value	B Coeff (95% CI)	Std. Coeff	*p*-value
SSS-Society	0.51 (0.27, 0.75)	< 0.001	**0.53 (0.29, 0.78)**	**0.12**	**< 0.001**	–	–	–
SSS-Community	0.61 (0.40, 0.81)	< 0.001	–	–	–	**0.58 (0.37, 0.78)**	**0.14**	**< 0.001**
Baseline MCS	0.35 (0.29, 0.41)	< 0.001	**0.32 (0.26, 0.38)**	**0.28**	**< 0.001**	**0.32 (0.26, 0.37)**	**0.28**	**< 0.001**
Demographics								
Age	0.03 (− 0.06, 0.11)	0.527	–	–	–	–	–	–
Education		0.995			**0.970**			**0.897**
No schooling	1.00		**1.00**			**1.00**		
At least some primary school	−0.08 (−1.06, 0.90)		**−0.09 (−1.09, 0.90)**	**− 0.01**		**0.20 (− 0.77, 1.16)**	**0.01**	
At least some secondary school	0.04 (−1.36, 1.45)		**−0.11 (−1.56, 1.35)**	**− 0.00**		**0.40 (− 0.99, 1.79)**	**0.02**	
At least some university/ college	0.08 (−1.69, 1.85)		**−0.46 (− 2.29, 1.37)**	**− 0.01**		**0.59 (− 1.17, 2.34)**	**0.02**	
Currently married	1.21 (0.36, 2.06)	0.005	**1.39 (0.51, 2.27)**	**0.08**	**0.002**	**1.30 (0.45, 2.15)**	**0.08**	**0.003**
Well-being factors								
PASE Score (IQR)	0.57 (−0.42, 1.56)	0.257	–	–	–	–	–	–
Currently living alone	1.42 (0.34, 2.49)	0.010	0.70 (− 0.59, 2.00)	0.03	0.287	0.80 (− 0.44, 2.03)	0.04	0.208
Medical history								
Diabetes	0.82 (−0.44, 2.07)	0.201	–	–	–	–	–	–
Hyperthyroidism	−2.87 (− 4.82, − 0.91)	0.004	−1.62 (−3.70, 0.47)	−0.04	0.129	−1.41 (−3.45, 0.63)	− 0.04	0.174
Hypothyroidism	−3.00 (−5.74, − 0.26)	0.032	**−3.00 (− 5.76, − 0.25)**	**−0.06**	**0.032**	**−2.77 (− 5.45, − 0.09)**	**−0.05**	**0.043**
Osteoporosis	−0.66 (− 2.18, 0.85)	0.392	–	–	–	–	–	–
History of stroke	−4.48 (−6.86, − 2.11)	< 0.001	**−3.24 (− 5.68, − 0.79)**	**−0.07**	**0.010**	**−4.19 (− 6.52, − 1.85)**	**−0.09**	**< 0.001**
Hypertension	0.06 (−0.79, 0.91)	0.893	–	–	–	–	–	–
CVD conditions	−1.63 (− 2.76, − 0.49)	0.005	**−1.48 (− 2.62, − 0.34)**	**− 0.07**	**0.011**	**−1.30 (− 2.41, − 0.18)**	**−0.06**	**0.023**
COPD	−1.08 (− 3.07, 0.91)	0.287	–	–	–	–	–	–
Current alcohol use	1.48 (− 1.08, 4.04)	0.258	–	–	–	–	–	–
Ever smoked	−0.30 (− 1.81, 1..20)	0.691	–	–	–	–	–	–
History of starvation	−0.89 (− 1.76, − 0.03)	0.043	−0.75 (−1.65, 0.15)	0.05	0.102	−0.62 (−1.49, 0.26)	−0.04	0.166
BMI > = 30	1.96 (−0.33, 4.25)	0.094	1.70 (−0.61, 4.02)	0.04	0.149	1.24 (−1.05, 3.53)	0.03	0.288
Walking speed (m/sec)	0.58 (−1.51, 2.67)	0.588	**–**	**–**	**–**	**–**	**–**	**–**

*SSS* Subjective social status, *MCS* Mental Component Score, *PASE* Physical Activity Scale for Elderly, *IQR* Interquartile range, *CVD* Cardiovascular diseases, *COPD* Chronic obstructive pulmonary disease, *BMI* Body mass index. Values in bold represent those variables retained in the final model. Values not bolded but shown in the final model column represent their value before being removed from the model

SSS-Society model: VIF range 1.006 to 1.366, R square 0.122

SSS-Community model: VIF range 1.005 to 1.358, R square 0.132

For males, lower SSS-Society was a significant predictor of decline in year-4 MCS (β_unstandardized_ = 0.29, 95% CI: 0.12, 0.47; β_standardized_ = 0.08), while SSS-Community did not show association. For females, lower scores in both SSS variables were significant predictors in decline in year-4 MCS models (SSS-Society β_unstandardized_ = 0.53, 95% CI: 0.29, 0.78; β_standardized_ = 0.12; SSS-Community β_unstandardized_ = 0.58, 95% CI: 0.37, 0.78; β_standardized_ = 0.14). Furthermore, when both SSS-Society and SSS-Community were simultaneously entered as candidate variables into a multivariable MCS model for the female sample, both SSS remained statistically significant, with SSS-Community having a stronger effect size (β_standardized_ = 0.12) compared with SSS-Society (β_standardized_ = 0.07). In all multivariable MCS models, aside from a lower baseline MCS score, history of stroke was a significant predictor of lower year-4 MCS.

### Model fit

All the models’ residuals were approximately normally distributed, and according to scatter plots of residuals vs. predicted values, there were no associations between residuals and predicted values and the residuals showed constant variance across predicted value. All final models reported a low VIF range (1.001 to 1.369).

## Discussion

This study examined long-term changes in HRQOL in older Chinese adults. Consistent across all models, baseline physical/mental component scores were the strongest predictors of their corresponding follow-up scores, indicating that physical and mental functioning measures have very robust influence across time. Our study noted that low SSS scores were associated with long term decline in various HRQOL indicators, confirming past research from other areas of the world [30, 31]. However, past studies did not include information about chronic health conditions. Our study adds to the literature by showing as these associations with SSS variables remained significant even after adjusting for educational attainment, socio-demographic factors, lifestyle variables, and a large number of health conditions, SSS variables have additional explanatory power for predicting HRQOL changes in older Chinese adults in Hong Kong. These SSS variables were shown to have standardized effect sizes similar to or greater than many baseline health variables retained in the final models. Lastly, we noted that a variety of health conditions (e.g. history of stroke in males, diabetes in females) and physical parameters (e.g. walking speed) rather than lifestyle factors such as alcohol consumption were also shown to be important predictors of long-term HRQOL changes in this older age population.

Our study noted marked differences between SSS variables and HRQOL changes between males and females, suggesting that pathways by which SSS influences HRQOL in older age differ between sexes in our urban Chinese study population. SSS-Society was significantly associated with changes in PCS for females but not males. Nonetheless, lower baseline SSS-Society was associated with greater declines in mental functioning in both sexes in our study. These findings contrast with the German follow-up study that noted that SSS-Society was not associated with changes in MCS [30]. SSS-Society makes explicit reference to concrete aspects of status such as income and occupation is therefore more likely to capture a mental average of lifetime access to material resources [24]. In Hong Kong, social welfare is poorly developed and there is limited assistance for medical care beyond basic primary care services [47]. Consequently, low financial/material resources can translate to much greater levels of deprivation than in Germany. Past research has pointed to income inequality being a source of mental illness and distress [48]. Since Hong Kong is noted to have extremely high income inequality and less social assistance, Hong Kong is likely to have worse mental health for those in the lowest social strata [49]. For females in our study, the cumulative life course effects of material and environmental conditions in a society with wide SES disparities appear to exert long-term effects on both physical and mental functioning. In the SSS-Society models, only baseline health conditions and walking speed were predictive of follow-up physical functioning for males. It is possible that while the perception of lower SSS at the societal level has effects on mental HRQOL in Hong Kong males, that physical functioning in males is much more resilient to these perceptions of lower rank in society at large. The Indonesian and German longitudinal studies noted that SSS-Society were significant predictors of physical health but did not stratify their results by sex [30, 31]. In order to compare our findings, we conducted a post-hoc analysis that did not stratify by sex and noted that SSS-Society was also a significant predictor of PCS in our Chinese sample (untabulated).

In contrast to SSS-Society, lower baseline SSS-Community score was an independent predictor of lower year-4 physical functioning among males. These results suggest that comparisons with one’s peer group (rather than society at large) exerts effects on long-term physical functioning in males. Pathways that have been proposed for SSS effects on physical health have included neuroendocrine effects from stress such as elevated cortisol levels [22]. It is also possible that there is reverse causation whereby males with good physical functioning perceive themselves to have greater social status than their peers who have poorer physical functioning. This is supported by post-hoc analysis which found baseline PCS to be a significant predictor of SSS-Community score among males (untabulated).

For females, in addition to comparisons with society at large, SSS-Community showed strong effects on long-term changes in mental functioning while these effects were of marginal significance for males. SSS-Community, which asks respondents to self-define social status, is likely to have greater influence from less tangible aspects of status such as social engagement, social capital, and peer esteem than SSS-Society, which has been speculated in previous literature [23, 50]. The self-defined communities may include not only friends but also members of community groups such as church groups. Hence, strategies to improve social engagement and increase social capital within one’s community may improve long-term mental HRQOL in females. Our findings were largely consistent with a meta-analysis which found that higher self-rating on a community ladder to be more strongly associated with mental health for older-aged samples than in younger populations [21]. The consistency of our findings with meta-analyses of cross-sectional studies call for policy considerations in building stronger communities within the older adult population to improve their quality of life. Since SSS-Community was significantly associated with MCS in females and PCS in males in our study, future research seeking to compare their findings should examine the effects of SSS on each sex separately. The generalizability of SSS-Community beyond Chinese populations to other East Asian cultures is unclear as it has been shown that there exists considerable heterogeneity in the sources of self-perceived social status in various Asian cultures [51]. Future studies should therefore be conducted in different countries.

This study had a number of limitations. First, analysis examined SES through educational attainment. Income was not included in the analysis as a measure of SES due to the fact that nearly all females were housewives who did not report personal income. Moreover, the most recent income of male participants was difficult to compare since they had been retired for varying lengths of time, ranging from a few months to several decades at the commencement of the study. Since the SSS-Society variable explicitly asks for respondents to rate themselves in accordance to income and jobs, this variable should therefore partially compensate for the non-inclusion of income in the final results. Furthermore, a previous study found SSS-Society to additionally be determined by household income, satisfaction with standard of living, and feeling of financial security regarding the future [52]. These additional factors support using the measure among homemakers who did not report a previous personal income. However, inclusion of indicators such as income, home ownership, or pre-retirement occupation would have likely reduced the explanatory power of the SSS-Society variable, which has been shown previously [31]. Therefore, the size of our findings must be taken into consideration. Our study, similar to the general Hong Kong population of that age, largely consisted of low education adults. However, there was a wide range of income levels among those who did report an income in the study sample (ranging from no income to a very high income in 2001 of $7700 USD/month, and an average income of $2250 USD/month). Secondly, our study sample consisting of ambulatory older adults may present some selection bias for healthier participants. Additionally, we should note that the data used for this study commenced in 2001 with a 4 year follow-up. However, we feel the relationship between SSS and HRQOL found in this study would continue to be applicable with the current time. Due to the fact that there have not been major societal changes for this age group during this period. The healthcare system in Hong Kong is largely unchanged [47, 53]. Furthermore, we found the GINI coefficient, a measure of societal inequality, has remained stable, suggesting inequalities have persisted over these years [37]. Nonetheless, the study had noted a wide range across various health indicators such as walking speed, chronic health conditions and interference with activities of daily living. Further studies may, however, be needed to generalize to less healthy and possibly institutionalized respondents. The study possessed a relatively large sample size of 2934 participants and a long-term follow-up of 4 years. Since approximately 15% of the original sample had died before year-4 follow-up, the attrition among those still alive at year 4 was only 8%. Unlike many previous studies, our study nonetheless, was able to include a comprehensive range of potential confounding factors that included a wide array of health conditions as well as lifestyle factors. Future studies that examine the relationship between SSS and HRQOL changes in older age populations should include SES variables relevant to retired populations such as home ownership or occupation before retirement. Moreover, since social capital and SSS have been recently shown to be correlated in East Asia [51], future studies may consider exploring social capital as a potential mediator on the relationship between SSS and HRQOL.

## Conclusion

Our study confirms and adds to previous literature of longitudinal associations between subjective social status and health-related quality of life, being the first to establish the association among older adults in an urban East Asian setting. Both SSS measures should be considered to be included in psychosocial batteries and surveys to predict decline in health-related quality of life measurements in older Chinese adults. Given that SSS-Society and SSS-Community show associations with long-term HRQOL, strategies to address both measures are warranted. For SSS-Society, which implicitly includes not only factors like income and occupation, but also other SES measures, addressing other forms of material resources such as housing may need to be improved. In SSS-Community, which is theorized to capture less tangible aspects of social status (peer esteem, social interaction), improvements in increasing community-level social engagement within communities should be considered by policymakers.

## Data Availability

Data is available upon request by emailing the corresponding author.

## Abbreviations

QOL: Quality of life
HRQOL: Health-related quality of life
SES: Socio-economic status
SSS: Subjective social status
PCS: Physical component score
MCS: Mental component score
